# Determining the association between repeatedly elevated serum gamma‐glutamyltransferase levels and risk of respiratory cancer: A nationwide population‐based cohort study

**DOI:** 10.1002/cam4.3735

**Published:** 2021-02-26

**Authors:** Ye Jin Lee, Kyung‐Do Han, Da Hye Kim, Chang‐Hoon Lee

**Affiliations:** ^1^ Division of Pulmonary, Allergy and Critical Care Medicine Department of Internal Medicine Kang Dong Sacred Heart Hospital Seoul Republic of Korea; ^2^ Department of Statistics and Actuarial Science Soongsil University Seoul Republic of Korea; ^3^ Department of Biostatistics College of Medicine Catholic University of Korea Seoul Republic of Korea; ^4^ Department of Internal Medicine Seoul National University College of Medicine Seoul National University Hospital Seoul Republic of Korea

**Keywords:** gamma‐glutamyltransferase, Korean health screening, laryngeal cancer, lung cancer, respiratory cancer

## Abstract

**Background:**

Although elevated serum gamma‐glutamyltransferase (GGT) is a known indicator of increased risk of several cancers, the clinical value of repeated measurements of GGT has not been determined. Therefore, we aimed to investigate whether repeatedly elevated serum GGT levels are associated with the risk of respiratory cancer incidence.

**Methods:**

We included participants who had undergone the Korean Health screening four times during 2009–2012 and had previously undergone four consecutive examinations. Those who were diagnosed with respiratory cancer before the date of examination were excluded. The participants obtained one GGT point if their GGT levels were in the highest quartile (the quartile 4 group). We analyzed the association between GGT points and respiratory cancer incidence by Cox proportional hazard models.

**Results:**

During mean follow‐up of 6.39 ± 1.2 years, 3,559,109 participants were enrolled. Of them, 8,944 (0.34%) men and 1,484 (0.14%) women were newly diagnosed with respiratory cancer. In multivariate analysis adjusted for confounding factors, male participants with 4 GGT points had a significantly higher hazards of developing respiratory cancer than those with 0 GGT points (hazard ratio [HR]: 1.39; 95% confidence interval [CI]: 1.31–1.48). Among female, participants with the highest points of GGT also had sixfold increased risk of developing laryngeal cancer. However, no significant association was observed between GGT points and lung cancer incidence among women (HR: 0.95; 95% CI: 0.81–1.11).

**Conclusion:**

Repeatedly elevated serum levels of GGT were associated with a higher risk of respiratory cancer incidence, especially in men. This finding suggests that physicians can identify a person with a higher risk of respiratory cancer through a simple repeated measurement of GGT.

## Graphical abstract

This study is first study to investigate whether repeatedly elevated Gamma‐glutamyltransferase (GGT) levels are associated with the risk of respiratory cancers including lung and laryngeal cancers using a large database. Among men, the higher the GGT points, the higher was the risk of respiratory cancer, both laryngeal and lung cancer development. Among women, elevated level of GGT is associated with sixfold higher risk of laryngeal cancer incidence. Persistently elevated serum levels of GGT are associated with an increased risk of respiratory cancer development.

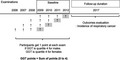

## 1 INTRODUCTION

Gamma glutamyltransferase (GGT) is distributed on cell membranes and abundant in tissues with a transport function, such as the kidney and biliary system.^1^ Elevated serum GGT is known as a marker of not only hepatic injury and alcohol consumption, but also various other disease including diabetes, cardiovascular disease, or metabolic syndrome.^2^, ^3^, ^4^ Several studies evaluated the mechanism underlying this association based on GGT’s protective role against inflammation and oxidative stress.^5^, ^6^ Inflammation and oxidative stress are established risk factors for cancer. Several epidemiological studies have reported the association with elevated serum GGT and the risk of cancer incidence.^6^, ^7^, ^8^ In an Austrian cohort study,^7^, ^8^ dose‐proportion to baseline GGT levels was significantly associated with respiratory and intrathoracic organ cancer. Mok et al. ^9^ showed that various cancers including lung cancer were related to the highest GGT quintiles using the insurance database. According to a previous biochemical study, non‐small cell lung carcinomas (NSCLCs) have higher GGT activity than normal lung tissues.^10^ However, these studies have limitations in that they used only one value of GGT such as the baseline level ^9^ or the sum of multiple measured GGT levels.^7^, ^8^ The GGT level may fluctuate, with certain patients having consistently elevated GGT levels, while others having isolated elevations. Therefore, studies based on a single measurement of GGT could not truly reflect and predict significance for respiratory system cancer incidence. Thus, serial measurement of GGT levels may result in higher sensitivity to evaluate the contributable risk of GGT levels in respiratory cancer than single measurement of GGT levels based on the fact that there is a lesser possibility to miss out the isolated elevation of GGT. Therefore, we aimed to investigate whether repeatedly elevated GGT levels are associated with the risk of respiratory cancers including lung and laryngeal cancers using a large database.

## 2 METHODS

### 2.1 Data source of National Health Insurance Services

We used the database of the National Health Insurance Service (NHIS), which covers 97.2% of the Korean population.^11^ All insured Koreans older than 40 years are eligible for health screening, which is performed every 2 years. The NHIS supports annual health check‐up for employees older than 20 years. The key variables of the NHIS maintain information on participants’ demographics, income, laboratory results, risk factors of health problems, claims for disease diagnosis codes of the International Classification of Diseases (ICD‐10), and treatment.^12^

This study protocol conformed to the ethical guidelines of the 1975 Declaration of Helsinki and was approved by the Institutional Review Board of Seoul National University Hospital (IRB No: 1906–009–1036). The requirement for informed consent from participants was waived because only de‐identified database entries were accessed for analytical purposes.

### 2.2 Study population

We included individuals aged ≥20 years who previously underwent health screening four times from 2005 until 2012 including the last examination performed between 2009 and 2012, which was defined as the baseline period in this study. Individuals with any existing cancer, as determined based on the ICD‐10 codes or expanding coverage for all cancers, at the baseline period were excluded. Individuals with missing data and those who died or had an event in 1 year were also excluded. Participants were followed up until December 2017.

### 2.3 Measurement of clinical parameters and biochemical analysis

Standardized self‐administered questionnaires were collected. The questionnaires comprised questions regarding patients’ age (years), sex, smoking status (never, ex, and current), alcohol consumption (frequency and amount), yearly income, level of physical activity, and underlying diseases.

Height (m) and body weight (kg) were measured using an electronic scale, and body mass index (BMI) was calculated as follows: BMI = body weight (kg)/height^2^ (m^2^). Waist circumference (WC) was measured at the midpoint between the lower costal margin and the iliac crest by a trained examiner. Systolic blood pressure (SBP) and diastolic blood pressure (DBP) were measured after 5 min of rest.

After overnight fasting, blood samples were collected from each participant and analyzed using standardized laboratory methods. Baseline laboratory examinations included fasting glucose, total cholesterol, low‐density lipoprotein cholesterol, high‐density lipoprotein cholesterol, triglycerides (TG), aspartate aminotransferase (AST), alanine aminotransferase (ALT), and GGT.

Diagnoses of hypertension (HTN), DM, and dyslipidemia were defined based on laboratory data or anthropometric measurements (SBP 140 mmHg or DBP 90 mmHg; fasting glucose level ≥126 mg/dL; total cholesterol levels ≥240 mg/dL) or ICD code (ICD codes I10–I13 or I15, E11–E14, and E78) and medication use including antihypertensive medications, insulin, or oral hypoglycemic agents or dyslipidemia medication.

**FIGURE 1:**
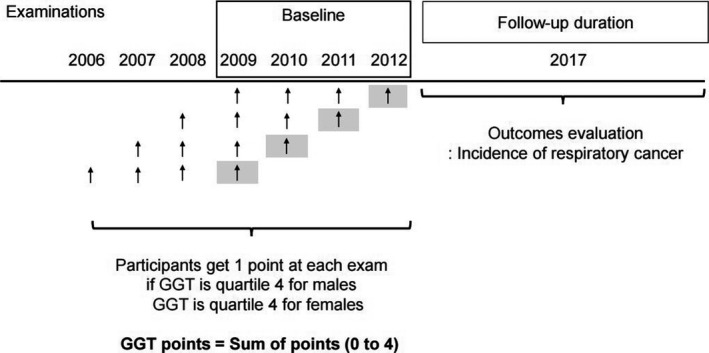
Flow of enrolment of the study participants

Participants were given “GGT points.” One point was given if the GGT levels were in the highest quartile (the quartile 4 group) on examination. An additional 1 point was given if the GGT level was in the quartile 4 group of the levels measured that year. For example, participants whose GGT level was repeatedly included in the highest quartile on four consecutive examinations (in 2010, 2011, 2012, and 2013) obtained 4 GGT points. By contrast, participants whose GGT level was not in the quartile 4 group in all four examinations obtained 0 GGT points (Figure 1).

### 2.4 Outcomes

We evaluated the incidence of respiratory cancer using the claims records of NHIS during the follow‐up period. Lung cancer was defined for ICD codes C33 or C34 with expanding benefit coverage for lung cancer. Laryngeal cancer was also defined using the ICD code C32 with the expanding benefit coverage for laryngeal cancer.

### 2.5 Statistical analysis

As there is a sex‐based difference in the levels of GGT,^13^ we conducted separate analyses by sex.

Continuous variables were expressed as mean ± standard deviation, while categorical variables were expressed as number and percentage. Group comparisons were performed using one‐way analysis of variance for continuous variables and chi‐square tests for categorical variables. For non‐normally distributed variables, log transformation was performed. The incidence rate of respiratory cancer was calculated as the number of events divided by the summation of person‐years (per 1,000). To adjust for covariates, multivariable Cox proportional hazards regression models were used. We also performed a subgroup analysis to evaluate the impact of GGT points on respiratory cancer incidence according to smoking status, BMI, baseline quartile of the GGT level, and alcohol consumption. Statistical analyses were performed using SAS version 9.3 (SAS Institute Inc.) and R version 3.2.3 (The R Foundation for Statistical Computing). A two‐sided *p* value of less than 0.05 was considered to indicate statistical significance.

## 3 RESULTS

### 3.1 Baseline characteristics

A total of 3,559,109 participants were included, and their baseline characteristics according to GGT points by sex are summarized in Table 1.

**TABLE 1:** Baseline characteristics according to the GGT points calculated as the sum of points at four consecutive exams. (A) Male subjects (B) Female subjects

	Total	GGT points				
(A)						
Male	N = 3,569,773	0 (n = 1,595,879)	1 (n = 260,084)	2 (n = 177,029)	3 (n = 181,702)	4 (n = 355,079)
Age, years	42.41 ± 10.53	41.64 ± 10.84	43.19 ± 10.47	43.31 ± 10.13	43.64 ± 9.82	44.19 ± 9.29
Smoking						
None	720,342 (28.03)	511,103 (32.03)	65,347 (25.13)	39,900 (22.54)	37,994 (20.91)	65,998 (18.59)
Ex	662,215 (25.77)	410,064 (25.7)	70,316 (27.04)	47,571 (26.87)	47,955 (26.39)	86,309 (24.31)
Current	1,187,216 (46.2)	674,712 (42.28)	124,421 (47.84)	89,558 (50.59)	95,753 (52.7)	202,772 (57.11)
Alcohol consumption						
None	720,354 (28.03)	540,201 (33.85)	64,798 (24.91)	36,701 (20.73)	32,092 (17.66)	46,562 (13.11)
Mild	1,583,612 (61.62)	952,992 (59.72)	165,116 (63.49)	114,805 (64.85)	118,787 (65.37)	231,912 (65.31)
Heavy	265,807 (10.34)	102,686 (6.43)	30,170 (11.6)	25,523 (14.42)	30,823 (16.96)	76,605 (21.57)
Regular PA	790,381 (30.76)	496,084 (31.09)	80,897 (31.1)	54,551 (30.81)	55,753 (30.68)	103,096 (29.03)
Lowest quartile of yearly income	399,370 (15.54)	244,182 (15.3)	42,615 (16.39)	28,596 (16.15)	28,873 (15.89)	55,104 (15.52)
BMI (kg/m^2^)	24.19 ± 3.01	23.55 ± 2.79	24.71 ± 2.91	25.1 ± 2.98	25.34 ± 3.03	25.64 ± 3.13
WC (cm)	83.14 ± 7.56	81.48 ± 7.16	84.46 ± 7.22	85.43 ± 7.25	86.08 ± 7.33	86.96 ± 7.45
Diabetes						
None	1,747,553 (68)	1,178,083 (73.82)	168,406 (64.75)	108,469 (61.27)	105,608 (58.12)	186,987 (52.66)
IFG	633,865 (24.67)	343,650 (21.53)	69,127 (26.58)	50,151 (28.33)	54,139 (29.8)	116,798 (32.89)
DM	188,355 (7.33)	74,146 (4.65)	22,551 (8.67)	18,409 (10.4)	21,955 (12.08)	51,294 (14.45)
Fasting glucose (mg/Dl)	97.2 ± 22.15	94.38 ± 18.25	98.47 ± 22.98	100.13 ± 24.85	101.84 ± 26.59	105.13 ± 29.72
Hypertension	543,619 (21.15)	249,986 (15.66)	63,584 (24.45)	48,861 (27.6)	55,827 (30.72)	125,361 (35.31)
SBP	123.78 ± 12.99	122 ± 12.45	124.88 ± 12.85	125.93 ± 13.02	126.8 ± 13.23	128.37 ± 13.68
DBP	77.98 ± 9.17	76.71 ± 8.79	78.75 ± 9.07	79.51 ± 9.16	80.19 ± 9.32	81.27 ± 9.6
Dyslipidemia	394,282 (15.34)	169,026 (10.59)	47,739 (18.36)	37,512 (21.19)	42,552 (23.42)	97,453 (27.45)
HDL (mg/Dl)	52.16 ± 13.63	52.38 ± 13.38	51.6 ± 13.93	51.57 ± 14.01	51.58 ± 13.9	52.18 ± 14.14
LDL (mg/Dl)	112.94 ± 32.32	112.28 ± 30.41	114.88 ± 33.13	114.76 ± 34.14	114.17 ± 35.16	112.95 ± 37.16
Total cholesterol (mg/Dl)	195.09 ± 34.85	190.25 ± 32.83	198.57 ± 34.96	201.16 ± 35.5	202.95 ± 36.32	207.29 ± 37.85
Triglyceride (mg/Dl)	129.81 (129.72–129.9)	112.69 (112.6–112.78)	141.48 (141.19–141.77)	154 (153.61–154.38)	164.57 (164.16–164.98)	187.2 (186.86–187.54)
CKD	97,299 (3.79)	61,043 (3.83)	10,196 (3.92)	6,888 (3.89)	6,863 (3.78)	12,309 (3.47)
GFR (Ml/min/1.73 m^2^)	88.86 ± 43.61	88.83 ± 44.55	88.57 ± 42.53	88.76 ± 43.04	88.97 ± 43.03	89.18 ± 40.64
LC	5771 (0.22)	2,175 (0.14)	728 (0.28)	556 (0.31)	627 (0.35)	1,685 (0.47)
Hepatitis	64,983 (2.53)	34,279 (2.15)	8,959 (3.44)	5,446 (3.08)	5,484 (3.02)	10,815 (3.05)
ALT (IU/L)	26.41 (26.4–26.43)	22.44 (22.43–22.46)	29.54 (29.49–29.6)	32.15 (32.07–32.22)	34.42 (34.34–34.51)	40.07 (40–40.14)
AST (IU/L)	25.9 (25.89–25.91)	23.64 (23.63–23.65)	27.11 (27.08–27.15)	28.47 (28.43–28.51)	29.84 (29.79–29.89)	33.52 (33.47–33.56)
GGT (IU/L)	36.3 (36.28–36.33)	24.79 (24.78–24.81)	43.21 (43.14–43.27)	53.64 (53.54–53.73)	66.15 (66.02–66.28)	107.44 (107.26–107.61)
(B)						
Female						
n	N = 989,336	0 (n = 566,518)	1 (n = 153,299)	2 (n = 83,247)	3 (n = 70,183)	4 (n = 116,089)
Age, years	41.61 ± 11.55	39.36 ± 11.04	42.51 ± 11.54	44.13 ± 11.57	45.34 ± 11.47	47.33 ± 10.9
Smoking						
None	950,040 (96.03)	547,422 (96.63)	147,238 (96.05)	79,376 (95.35)	66593 (94.88)	109411 (94.25)
Ex	16,273 (1.64)	8,976 (1.58)	2,493 (1.63)	1,454 (1.75)	1294 (1.84)	2056 (1.77)
Current	23,023 (2.33)	10,120 (1.79)	3,568 (2.33)	2,417 (2.9)	2296 (3.27)	4622 (3.98)
Alcohol consumption						
None	661,884 (66.9)	383,621 (67.72)	103,332 (67.41)	55,147 (66.25)	45838 (65.31)	73946 (63.7)
Mild	318,233 (32.17)	179,199 (31.63)	48,446 (31.6)	26,984 (32.41)	23345 (33.26)	40259 (34.68)
Heavy	9,219 (0.93)	3698 (0.65)	1,521 (0.99)	1,116 (1.34)	1000 (1.42)	1884 (1.62)
Regular PA	270,758 (27.37)	155,736 (27.49)	42,388 (27.65)	22,839 (27.44)	18923 (26.96)	30872 (26.59)
Lowest quartile of yearly income	365,612 (36.96)	181,102 (31.97)	61,625 (40.2)	35,775 (42.97)	31499 (44.88)	55611 (47.9)
BMI (kg/m^2^)	22.45 ± 3.22	21.79 ± 2.82	22.56 ± 3.15	23.14 ± 3.39	23.64 ± 3.53	24.36 ± 3.71
WC (cm)	73.52 ± 8.28	71.77 ± 7.42	73.86 ± 8.07	75.36 ± 8.49	76.6 ± 8.78	78.47 ± 9.09
Diabetes						
None	805,990 (81.47)	489,351 (86.38)	124,149 (80.98)	64,211 (77.13)	51316 (73.12)	76963 (66.3)
IFG	148,818 (15.04)	68,823 (12.15)	24,264 (15.83)	15,071 (18.1)	14042 (20.01)	26618 (22.93)
DM	34,528 (3.49)	8,344 (1.47)	4,886 (3.19)	3,965 (4.76)	4825 (6.87)	12508 (10.77)
Fasting glucose (mg/dL)	91.41 ± 16.31	89.21 ± 12.41	91.39 ± 15.32	93.13 ± 17.79	94.99 ± 20.38	98.8 ± 25.3
Hypertension	130,992 (13.24)	45,016 (7.95)	21,361 (13.93)	15,255 (18.32)	15700 (22.37)	33660 (28.99)
SBP	116.17 ± 13.59	114.11 ± 12.72	116.63 ± 13.55	118.33 ± 14.04	119.76 ± 14.23	121.91 ± 14.56
DBP	72.92 ± 9.26	71.68 ± 8.83	73.17 ± 9.25	74.2 ± 9.46	75.08 ± 9.54	76.39 ± 9.73
Dyslipidemia	128,796 (13.02)	45,810 (8.09)	21,112 (13.77)	14,928 (17.93)	15084 (21.49)	31862 (27.45)
HDL	60.82 ± 15.16	61.63 ± 14.7	60.57 ± 15.3	59.93 ± 15.73	59.3 ± 15.65	58.74 ± 16.1
LDL	110.02 ± 31.3	106.75 ± 29.16	111.38 ± 31.72	113.91 ± 33.05	115.34 ± 33.97	118.18 ± 35.21
Total cholesterol (mg/dL)	190.33 ± 34.94	185.48 ± 32.85	191.82 ± 35.03	195.68 ± 35.98	198.31 ± 36.74	203.37 ± 37.76
Triglyceride (mg/dL)	85.34 (85.25–85.42)	76.5 (76.4–76.59)	87.69 (87.47–87.91)	95.8 (95.47–96.13)	103.29 (102.89–103.69)	115.13 (114.78–115.49)
Chronic kidney disease	48,936 (4.95)	27,549 (4.86)	7,518 (4.9)	4,180 (5.02)	3570 (5.09)	6119 (5.27)
Liver cirrhosis	748 (0.08)	225 (0.04)	109 (0.07)	64 (0.08)	86 (0.12)	264 (0.23)
Hepatitis	20,457 (2.07)	9,414 (1.66)	3,632 (2.37)	2,066 (2.48)	1867 (2.66)	3478 (3)
GFR (mL/min/1.73 m^2^)	90.46 ± 32.28	91.31 ± 33.02	90.18 ± 32.08	89.63 ± 32.08	89.19 ± 31.83	88.01 ± 28.96
ALT	16.82 (16.81–16.84)	14.74 (14.73–14.76)	17.38 (17.34–17.41)	19.11 (19.05–19.17)	20.85 (20.78–20.92)	24.6 (24.53–24.67)
AST	21.38 (21.36–21.39)	20.06 (20.05–20.08)	21.64 (21.61–21.67)	22.58 (22.54–22.63)	23.6 (23.55–23.66)	25.95 (25.89–26)
GGT	17.53 (17.51–17.54)	13.52 (13.51–13.53)	18.15 (18.12–18.18)	21.94 (21.89–21.99)	26.28 (26.21–26.35)	39.57 (39.46–39.69)

Categorical variables are expressed as number (%); continuous variables are expressed mean ± standard deviation.

Abbreviations: ALT, alanine aminotransferase; AST, aspartate aminotransferase; BMI, body mass index; DBP, diastolic blood pressure; DM, diabetes mellitus; GFR, Glomerular Filtration Rate; GGT, Gamma‐glutamyltransferase; HDL, high‐density lipoprotein; IFG, impaired fasting glucose; LDL, low‐density lipoprotein; PA, physical activity; SBP, systolic blood pressure; WC, waist circumference.

Among men, 62.1% of the participants had 0 GGT points, 260,084 (10.1%) had one GGT point, 177,029 (6.9%) had 2 GGT points, 181,702 (7.1%) had 3 GGT points, and 355,079 (13.8%) had 4 GGT points. Those with higher GGT points were more likely to be current smokers and heavy drinkers and have metabolic disease including DM, HTN, and dyslipidemia. Those with higher GGT points had worse health indices including higher BMI, WC, SBP, DBP, fasting glucose, total cholesterol, and TG, ALT, and AST than those with lower GGT points. During a mean follow‐up of 6.39 ± 1.2 years, 8,944 (0.34%) participants with respiratory cancer were identified. Among them, 8,348 (0.32%) and 642 (0.02%) participants were diagnosed with lung and laryngeal cancers, respectively. Moreover, 46 (0.00001%) participants were diagnosed with both lung cancer and laryngeal cancer.

Among women, 566,518 (57.3%) had 0 GGT points, 153,299 (15.5%) had one GGT point, 83,247 (8.4%) had 2 GGT points, 70,183 (7.1%) had 3 GGT points, and 116,089 (11.7%) had 4 GGT points. Those with higher GGT points were more likely to be current smokers and heavy drinkers and have metabolic disease including DM, HTN, and dyslipidemia. Those with higher GGT points had worse health indices including higher BMI, WC, SBP, DBP, fasting glucose, total cholesterol, and TG, ALT, and AST than those with lower GGT points. A total of 1,484 (0.15%) participants had respiratory cancer, while 1,470 (0.15%) and 15 (0.002%) participants were diagnosed with lung and laryngeal cancers, respectively. Only one participant was diagnosed with both lung cancer and laryngeal cancer. In both sexes, the higher the GGT points, the higher was the BMI and WC. Furthermore, those with the highest GGT points were associated with increased levels of fasting glucose, BP, and TG, which are components of metabolic syndrome, compared with those with 0 GGT points.

We also evaluated the demographic and clinical characteristics of the study population according to baseline GGT quartiles by sex. Based on the results of the GGT point group, participants with higher baseline GGT quartiles showed worse health indices and were likely to have risk factors for health problems (Supplementary Table 1).

### 3.2 Association of GGT with the risk of incidence of lung and laryngeal cancers

Among men, the higher the GGT points, the higher was the risk of respiratory cancer (*p* for trend < 0.001). Those with 4 GGT points had the highest risk of respiratory cancers compared with those with 0 GGT points after adjustment for age, smoking status, alcohol consumption, income, HTN, DM, dyslipidemia, BMI, and physical activity (adjusted HR [aHR]: 1.39, 95% CI: 1.31–1.48). Participants with 4 GGT points had a higher risk of laryngeal cancer incidence than those with 0 GGT points even after adjustment for age, smoking status, alcohol consumption, income, HTN, DM, dyslipidemia, BMI, and physical activity (aHR: 1.87 95% CI:1.51–2.32). We also found that the higher the GGT points, the higher was the incidence of lung cancer (4 GGT points vs. 0 GGT point; aHR: 1.36, 95% CI: 1.27–1.45) (Table 2).

**TABLE 2:** Incidence of respiratory cancer including lung and laryngeal cancer according to the GGT points based on four consecutive examinations

GGT points	Male					Female				
	N	Events	IR^a^	Model 1 aHR^b^ (95% CI)	Model 2 aHR (95% CI)	N	Events	IR	Model 1 aHR (95% CI)	Model 2 aHR (95% CI)
	Respiratory cancer									
0	1,595,879	4,855	0.47	1 (ref.)	1 (ref.)	566,518	713	0.20	1 (ref.)	1 (ref.)
1	260,084	989	0.59	1.25 (1.17, 1.34)	1.15 (1.07, 1.23)	153,299	250	0.26	1.30 (1.13, 1.50)	1.00 (0.87,1.16)
2	177,029	725	0.66	1.35 (1.25, 1.46)	1.27 (1.17, 1.37)	83,247	132	0.25	1.27 (1.06, 1.53)	0.88 (0.73,1.06)
3	181,702	767	0.66	1.39 (1.29, 1.50)	1.30 (1.20, 1.40)	70,183	151	0.35	1.73 (1.45,2.07)	1.11 (0.93,1.33)
4	355,079	1,608	0.71	1.51 (1.43, 1.60)	1.39 (1.31, 1.48)	116,089	238	0.33	1.67 (1.44, 1.93)	0.96 (0.83,1.13)
*p* for trend				< 0.0001	< 0.0001				<.0001	0.86
	Laryngeal cancer									
0	1,595,879	293	0.03	1 (ref.)	1 (ref.)	566,518	3	0.0008	1 (ref.)	1 (ref.)
1	260,084	73	0.04	1.53 (1.18, 1.98)	1.33 (1.02, 1.72)	153,299	3	0.003	3.71 (0.75, 18.38)	2.86 (0.57, 14.34)
2	177,029	61	0.05	1.88 (1.43, 2.48)	1.62 (1.22, 2.15)	83,247	1	0.002	2.29 (0.24, 22.04)	1.55 (0.16, 15.23)
3	181,702	65	0.06	1.95 (1.49, 2.56)	1.63 (1.24, 2.15)	70,183	2	0.005	5.48 (0.92, 32.80)	3.53 (0.57, 21.94)
4	355,079	150	0.07	2.33 (1.91, 2.83)	1.87 (1.51, 2.32)	116,089	6	0.008	10.1 (2.52, 40.35)	5.97 (1.38, 25.91)
*p* for trend		642		<.0001	<.0001				0.0008	0.02
	Lung cancer									
0	1,595,879	4,587	0.44	1 (ref.)	1 (ref.)	566,518	710	0.20	1 (ref.)	1 (ref.)
1	260,084	922	0.55	1.23 (1.15,1.33)	1.14 (1.06, 1.22)	153,299	247	0.26	1.29 (1.12, 1.49)	0.99 (0.86, 1.16)
2	177,029	666	0.58	1.31 (1.21,1.42)	1.24 (1.14, 1.34)	83,247	131	0.25	1.27 (1.05, 1.53)	0.88 (0.73, 1.06)
3	181,702	707	0.60	1.36 (1.26, 1.47)	1.28 (1.18, 1.38)	70,183	149	0.34	1.72 (1.44, 2.05)	1.10 (0.92, 1.32)
4	355,079	1,466	0.65	1.46 (1.37, 1.54)	1.36 (1.27, 1.45)	116,089	233	0.32	1.64 (1.41, 1.90)	0.95 (0.81, 1.11)
*p* for trend				<.0001	<.0001				<.0001	0.72

Model 1 without any adjustments.

Model 2 adjusted for age, smoking status, alcohol habit, income, hypertension, dyslipidemia, diabetes, body mass index, and regular physical activity.

^a^ IR: incidence rates per 1000 person years.

^b^ aHR: adjusted hazard ratio.

Women with 4 GGT points had a sixfold higher risk of laryngeal cancer than those with 0 GGT points (aHR: 5.97, 95% CI: 1.38–25.91), and there was a significant dose–response relationship (*p* for trend = 0.02) (Table 2). However, no significant associations were observed between GGT points and respiratory cancers and lung cancer incidence.

In the analysis using baseline GGT quartiles, the highest baseline GGT quartile was significantly associated with increased respiratory cancer incidence, showing a dose–response relationship (*p* for trend across GGT quartiles, all *p* for trend < 0.001). This result was observed only among men (Table S2).

**FIGURE 2:**
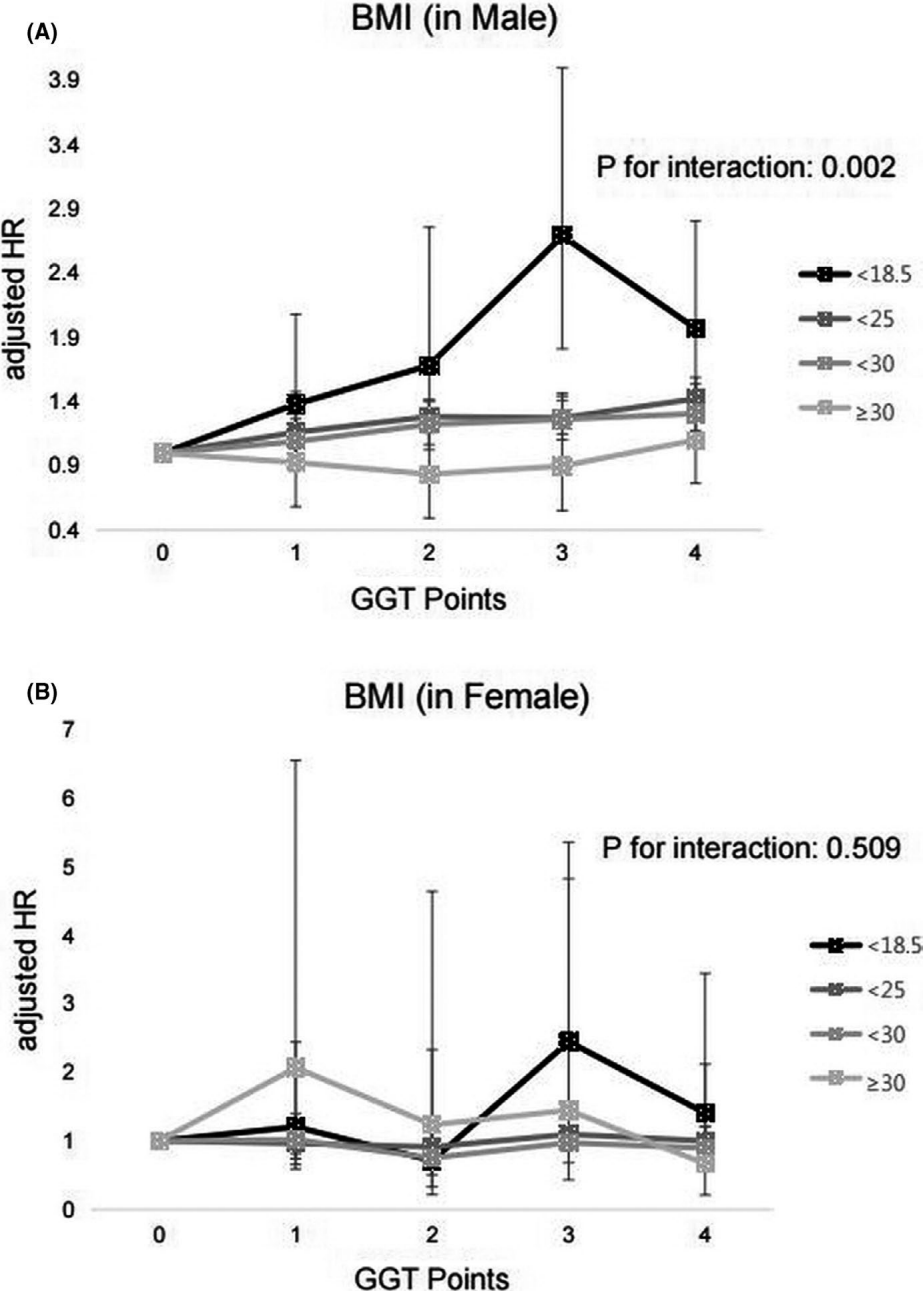
Association of the GGT points and risk of respiratory cancer, based on BMI levels. (A) Male (B) Female

Subgroup analysis was performed based on smoking status, BMI, baseline GGT quartile group, and alcohol consumption (Table S3). Among men, significant effect modification by BMI categories was observed. Among men with BMI lower than 18.5 kg/m^2^, those with higher GGT levels (3 and 4 GGT points) had significantly higher risk of respiratory cancer (aHR: 2.69, 95% CI: 1.81–4.00; aHR: 1.97, 95% CI: 1.38–2.81, respectively) than those with lower GGT levels (0 to 2 GGT points) (*p* for interaction = 0.002) (Figure 2). No significant interactions were observed in other subgroups. Among women, participants with BMI lower than 18.5 kg/m^2^ and higher GGT levels (3 and 4 GGT points) had a higher risk of respiratory cancer than those with lower GGT levels, but there was no significant difference (Figure 2).

## 4 DISCUSSION

This population‐based, large‐scale study reported that persistently elevated serum levels of GGT are associated with an increased risk of respiratory cancer development. Among men, persistently elevated serum levels of GGT were associated with increased respiratory cancer incidence, in both lung and laryngeal cancers; among women, GGT level was associated with only increased incidence of laryngeal cancer, but not with lung cancer. Our study had sufficient power due to the large sample size, and we analyzed men and women separately, which helped us observe the influence of sex‐based differences on the association between GGT and respiratory cancer risk. Furthermore, the long follow‐up duration (64.8 months) does not add value to this study because the outcome (cancer incidence) does not occur within a short period. We included a sufficiently long washout period (4 year), which allowed us to determine the true association between GGT elevation and risk of respiratory cancer by excluding participants with respiratory cancer before elevated GGT exposure.

We used serial measurement of GGT, not just single measurement (baseline). No study has previously reported the association between repeated elevation of GGT levels and the increased risk of respiratory cancer incidence. Previous epidemiological studies have evaluated the overall cancer incidence by performing a single GGT measurement or other study that performed a series of GGT measurements and they contained and analyzed in two groups, one group included participants whose the mean of the multiple GGT measurements was higher than the baseline, the other group was not.^7^, ^8^, ^9^ In our study, 13.8% of men and 11.7% of women had 4 GGT points. Thus, if repeated measurements of GGT were not performed, many participants would have been diagnosed as negative. Those with 1–3 GGT points might have a normal or low GGT level when serum GGT is measured only at one time point. Furthermore, our study showed that not only participants with 2–4 GGT points but also those with one GGT point had increased risk of respiratory cancer compared with those with 0 GGT points in both sexes. In men, the higher the GGT points, the higher was the risk of respiratory cancer. This finding suggests that repetitive and sequential measurement of the serum GGT enables the identification of groups with higher risk for respiratory cancer.

The relationship between the persistently elevated GGT level and the incidence of respiratory cancers can be explained by several hypotheses. First, persistently elevated serum GGT levels reflect chronic inflammation and oxidative stress, which contribute to tumor development and progression.^14^, ^15^ Prolonged production of cytokines and growth factors from alveolar macrophages and lymphocytes by chronic inflammation was observed in lung cancer patients.^16^, ^17^ Furthermore, hydrogen peroxide (H_2_O_2_), a highly reactive oxygen species known to inhibit the growth of lung cancer, is metabolized into O_2_ and H_2_O by glutathione peroxidase; previous studies have reported that the association of GSH level with lung cancer is likely through this mechanism.^18^ However, no study has explored the mechanism of how elevated levels of both GSH and GGT are related to lung cancer, but several studies have shown that they were elevated in NSCLC cell line and in the tumor‐bearing lobe.^10^, ^19^, ^20^ Second, an elevated GGT level is related to obesity,^21^, ^22^ which is a known risk factor of developing lung cancer.^23^ Our study showed that the higher the GGT points, the higher the BMI and WC. Moreover, a persistently elevated GGT has a stronger association with obesity. Those with higher serum GGT levels are more likely to have metabolic syndrome, which increases the incidence of laryngeal cancer.^24^ We also found that as GGT points increased, the levels of fasting glucose, BP, and TG, which are components of metabolic syndrome also increased.

Our study did not observe any significant relationship between GGT levels and the risk of total respiratory cancer and lung cancer in women.^8^, ^9^ In fact, previous studies showed contrasting results. In the Austrian cohort study, elevated GGT concentrations were associated with an increased risk of malignant neoplasms of the respiratory system/intrathoracic organ among women. However, the Austrian study included mesothelioma; thymoma; and malignant neoplasm of the nasal cavities, middle ear, larynx, trachea, and lung; and heart tumors to define “tumors of the respiratory system and intrathoracic organ”.^8^ Whether serum GGT level is associated with an increased risk of lung cancer incidence alone remains unclear. Another study that analyzed the association between baseline GGT level and lung cancer incidence in women showed no significant relationship between two variables.^9^ By contrast, our study have advantages; that is, we analyzed the incidence of cancer in each organ, instead of showing the sum of organ‐specific cancer, such as “respiratory system and intrathoracic organs” and repeated measurements of GGT not at the baseline.” The differences in histological subtypes of tumors between sexes could affect the results. Women are more likely to present adenocarcinoma histologic type than men.^25^ However, we could not verify the hypothesis due to lack of data in our study. Lastly, the lower cutoff levels of the highest quartile in women than in men might mitigate the effects of GGT levels on cancer incidences because the absolute GGT levels were lower in women than in men. In our study, the average GGT level in the highest quartile (Q4) in women was ≥21, while that in men was ≥50, which is more than twice as different as that in women.

GGT levels are influenced by age, dietary, and lifestyle‐related factors.^26^, ^27^ A subgroup analysis was performed to determine the influence of these factors. We found significant interactions between the BMI group and the effects of GGT points on the risk of total respiratory cancer, lung cancer, and laryngeal cancer in men (*p* for interactions < 0.05). Lean participants with a BMI of <18.5 kg/m^2^ showed the highest association between the GGT points and the risk of cancers. If both GGT and adiposity increased the risk of respiratory cancers, GGT could have more room for affecting the risk of cancer incidence in the group with low BMI where the effect of adiposity is minimal.

In conclusion, repeatedly elevated serum levels of GGT were associated with a higher risk of respiratory cancer incidence, especially in men, in our large population‐based cohort study. Physicians could identify a person with higher risk of respiratory cancer by conducting a simple repeated measurement of GGT levels.

## CONFLICT OF INTEREST

The authors made no disclosures.

## AUTHOR CONTRIBUTIONS

YJL: Conceptualization, Methodology, Writing‐ Original draft preparation, Validation. KDH and DHK: Conceptualization, Data curation, Software, Validation. CHL: Conceptualization, Writing‐ Original draft preparation, Writing‐ Reviewing and Editing, Supervision.

## 5 DATA AVAILABLE STATEMENT

The data that support the findings of this study are available on request from the corresponding author. The data are not publicly available due to privacy or ethical restrictions.

## Supporting information

Table S1‐S3
