# Supplementary material for: Determining the association between repeatedly elevated serum gamma‐glutamyltransferase levels and risk of respiratory cancer: A nationwide population‐based cohort study
Source: Cancer Med. 2021 Feb 26;10(4):1366–76. doi: 10.1002/cam4.3735 (PMC7925999; doi:10.1002/cam4.3735)
Supplement: Supplementary file 1 — Table S1‐S3 [file CAM4-10-1366-s001.docx]

**Supplementary Table 1.** Baseline characteristics according to the baseline GGT quantile group. (A) Male subjects (B) Female subjects

(A)

|  | Baseline GGT | | | |
| --- | --- | --- | --- | --- |
| Male | Q1 (n=657,683) | Q2 (n=618,761) | Q3 (n=647,669) | Q4 (n=645,660) |
| AGE | 40.98±11.09 | 42.39±10.81 | 42.98±10.31 | 43.29±9.7 |
| SMOKING |  |  |  |  |
| None | 241,204(36.67) | 186,440(30.13) | 162,994(25.17) | 129,704(20.09) |
| Ex | 165,234(25.12) | 163,938(26.49) | 172,250(26.6) | 160,793(24.9) |
| Current | 251,245(38.2) | 268,383(43.37) | 312,425(48.24) | 355,163(55.01) |
| Alcohol consumption |  |  |  |  |
| None | 267,422(40.66) | 197,253(31.88) | 158,113(24.41) | 97,566(15.11) |
| Mild | 365,010(55.5) | 379,099(61.27) | 418,877(64.67) | 420,626(65.15) |
| Heavy | 25,251(3.84) | 42,409(6.85) | 70,679(10.91) | 127,468(19.74) |
| Regular PA | 215,019(32.69) | 194,731(31.47) | 196,405(30.32) | 184,226(28.53) |
| Lowest quartile yearly income | 102,886(15.64) | 97,255(15.72) | 99,625(15.38) | 99,604(15.43) |
| BMI (kg/m^2^) | 22.78±2.55 | 23.8±2.74 | 24.73±2.9 | 25.46±3.1 |
| WC (cm) | 79.46±6.69 | 82.16±6.99 | 84.51±7.2 | 86.44±7.44 |
| Diabetes |  |  |  |  |
| None | 509,466(77.46) | 446,631(72.18) | 427,138(65.95) | 364,318(56.43) |
| IFG | 122,566(18.64) | 138,222(22.34) | 169,678(26.2) | 203,399(31.5) |
| DM | 25,651(3.9) | 33,908(5.48) | 50,853(7.85) | 77,943(12.07) |
| Fasting glucose (mg/dL) | 92.84±16.68 | 95.09±19.26 | 98.01±22.58 | 102.86±27.32 |
| Hypertension | 79,372(12.07) | 108,251(17.49) | 151,102(23.33) | 204,894(31.73) |
| SBP (mmHg) | 120.3±12.17 | 122.55±12.45 | 124.67±12.74 | 127.6±13.43 |
| DBP (mmHg) | 75.44±8.59 | 77.1±8.76 | 78.66±8.98 | 80.75±9.45 |
| Dyslipidemia | 43,374(6.59) | 74,156(11.98) | 113,984(17.6) | 162,768(25.21) |
| HDL | 52.96±13.32 | 52.03±13.49 | 51.33±13.56 | 52.31±14.1 |
| LDL | 107.57±28.39 | 114.06±30.59 | 116.35±32.73 | 113.92±36.37 |
| Total cholesterol (mg/dL) | 182.03±30.75 | 192.63±32.37 | 199.53±34.02 | 206.31±37.15 |
| Triglyceride (mg/dL) | 96.17(96.06-96.28) | 117.9(117.75-118.05) | 141.26(141.08-141.44) | 177.52(177.29-177.76) |
| Chronic kidney disease | 24,774(3.77) | 23,704(3.83) | 25,262(3.9) | 23,559(3.65) |
| GFR | 89.73±44.53 | 88.44±43.2 | 88.28±44.39 | 88.96±42.24 |
| Liver cirrhosis | 947(0.14) | 1,111(0.18) | 1317(0.2) | 2,396(0.37) |
| Hepatitis | 15,319(2.33) | 14,611(2.36) | 15,907(2.46) | 19,146(2.97) |
| ALT* | 18.89(18.87-18.91) | 23.08(23.06-23.1) | 28.48(28.45-28.51) | 39.22(39.17-39.27) |
| AST* | 22.03(22.02-22.05) | 23.79(23.78-23.81) | 26.24(26.22-26.26) | 32.71(32.68-32.74) |
| GGT | 17.36(17.35-17.37) | 27.0 (26.99-27.01) | 40.93(40.91-40.94) | 90.66(90.56-90.76) |

(B)

|  | Baseline GGT | | | |
| --- | --- | --- | --- | --- |
| Female | Q1 (n=219,583) | Q2 (n=294,048) | Q3 (n=221,623) | Q4 (n=254,082) |
| Age | 38.02±10.63 | 40.23±11.23 | 42.51±11.59 | 45.54±11.34 |
| Smoking |  |  |  |  |
| None | 212,816(96.92) | 283,917(96.55) | 212,500(95.88) | 240,807(94.78) |
| Ex | 3,712(1.69) | 4,519(1.54) | 3,609(1.63) | 4,433(1.74) |
| Current | 3,055(1.39) | 5,612(1.91) | 5,514(2.49) | 8,842(3.48) |
| Alcohol consumption |  |  |  |  |
| None | 15,6010(71.05) | 197,955(67.32) | 144,706(65.29) | 163,213(64.24) |
| Mild | 62,647(28.53) | 94,166(32.02) | 74,708(33.71) | 86,712(34.13) |
| Heavy | 926(0.42) | 1,927(0.66) | 2,209(1) | 4,157(1.64) |
| Regular PA | 62,110(28.29) | 81,015(27.55) | 60,417(27.26) | 67,216(26.45) |
| Lowest quartile of yearly income | 64,793(29.51) | 100,725(34.25) | 85,881(38.75) | 114,213(44.95) |
| BMI (kg/m^2­^) | 21.54±2.62 | 21.89±2.86 | 22.55±3.18 | 23.82±3.64 |
| WC | 71.16±7.19 | 72.06±7.47 | 73.79±8.09 | 77.04±8.97 |
| Diabetes |  |  |  |  |
| None | 193,217(87.99) | 251,890(85.66) | 179,634(81.05) | 181,249(71.33) |
| IFG | 23,506(10.7) | 36,808(12.52) | 34,829(15.72) | 53,675(21.13) |
| DM | 2,860(1.3) | 5,350(1.82) | 7,160(3.23) | 19,158(7.54) |
| Fasting glucose (mg/dL) | 88.46±11.73 | 89.54±12.91 | 91.44±15.43 | 96.11±21.97 |
| Hypertension | 13,237(6.03) | 26,957(9.17) | 31,041(14.01) | 59,757(23.52) |
| SBP (mmHg) | 112.97±12.28 | 114.59±12.9 | 116.73±13.56 | 120.27±14.38 |
| DBP (mmHg) | 70.91±8.61 | 71.97±8.92 | 73.27±9.22 | 75.44±9.63 |
| Dyslipidemia | 13,542(6.17) | 25577(8.7) | 30,603(13.81) | 59,074(23.25) |
| HDL (mg/Dl) | 61.58±14.9 | 61.44±14.68 | 60.75±14.95 | 59.5±15.98 |
| LDL (mg/Dl) | 103.11±27.76 | 107.7±29.22 | 112.19±31.52 | 116.8±34.59 |
| Total cholesterol (mg/dL) | 180.92±32.36 | 186.48±32.46 | 192.72±34.36 | 200.84±37.28 |
| Triglyceride (mg/dL) | 72.9(72.76-73.04) | 77.69(77.56-77.82) | 87.33(87.15-87.51) | 106.85(106.62-107.07) |
| Chronic kidney disease | 10,305(4.69) | 13,445(4.57) | 11,069(4.99) | 14,117(5.56) |
| GFR | 94.41±33.28 | 90.54±32.13 | 89.08±32.72 | 88.15±30.82 |
| Liver cirrhosis | 110(0.05) | 137(0.05) | 125(0.06) | 376(0.15) |
| Hepatitis | 3,858(1.76) | 5,284(1.8) | 4,326(1.95) | 6,989(2.75) |
| ALT | 13.61(13.59-13.63) | 14.93(14.92-14.95) | 16.92(16.9-16.95) | 23.07(23.02-23.11) |
| AST | 19.29(19.27-19.31) | 20.22(20.2-20.24) | 21.34(21.32-21.36) | 24.95(24.92-24.98) |
| GGT | 10.39(10.38-10.39) | 14.4(14.39-14.4) | 18.65(18.64-18.65) | 32.78(32.73-32.84) |

Supplementary Table 2. incidence of respiratory cancer including lung and laryngeal cancer according to the baseline GGT quantile group

| **Baseline GGT** | **Male** | | | | **Female** | | | |
| --- | --- | --- | --- | --- | --- | --- | --- | --- |
|  | N | Event | IR^*^ | aHR (95% CI) ^†^ ^*^ | N | Event | IR | aHR (95% CI) ^*^ |
| **Respiratory cancer** | | | | | | | | |
| Q1 | 657,683 | 1,758 | 0.42 | 1(ref.) | 219,583 | 231 | 0.17 | 1(ref.) |
| Q2 | 618,761 | 2,161 | 0.54 | 1.22 (1.14,1.30) | 294,048 | 423 | 0.23 | 1.12 (0.95,1.31) |
| Q3 | 647,669 | 2,368 | 0.57 | 1.30 (1.22,1.38) | 221,623 | 346 | 0.25 | 1.02 (0.86,1.20) |
| Q4 | 645,660 | 2,657 | 0.64 | 1.51 (1.41,1.61) | 254,082 | 484 | 0.31 | 1.04 (0.88,1.22) |
| p for trend |  | | | <.0001 |  | | | 0.84 |
| **Laryngeal cancer** | | | | | | | | |
| Q1 | 657,683 | 96 | 0.02 | 1(ref.) | 21,9583 | 3 | 0.002 | 1(ref.) |
| Q2 | 618,761 | 129 | 0.03 | 1.28 (0.98,1.67) | 294,048 | 0 | - | - |
| Q3 | 647,669 | 176 | 0.04 | 1.62 (1.25,2.10) | 221,623 | 4 | 0.003 | 0.88 (0.19,4.03) |
| Q4 | 645,660 | 241 | 0.06 | 2.15 (1.66,2.78) | 254,082 | 8 | 0.005 | 1.27 (0.31,5.19) |
| p for trend |  |  |  | <.0001 |  |  |  | 0.21 |
| **Lung cancer** | | | | | | | | |
| Q1 | 657,683 | 1,668 | 0.39 | 1(ref.) | 219,583 | 228 | 0.16 | 1(ref.) |
| Q2 | 618,761 | 2,044 | 0.51 | 1.22 (1.14,1.30) | 294,048 | 423 | 0.23 | 1.13 (0.96,1.33) |
| Q3 | 647,669 | 2,209 | 0.53 | 1.28 (1.20,1.37) | 221,623 | 342 | 0.25 | 1.02 (0.86,1.21) |
| Q4 | 645,660 | 2,427 | 0.59 | 1.46 (1.37,1.57) | 254,082 | 477 | 0.30 | 1.04 (0.88,1.22) |
| p for trend |  | | | <.0001 |  |  |  | 0.78 |

*IR: incidence rates per 1000 person years

† aHR: adjusted hazard ratio: adjusted for age, smoking status, alcohol consumption, income, hypertension, dyslipidemia, diabetes, body mass index, and regular physical activity

Supplementary Table 3. Subgroup analysis of incidence of respiratory cancer in (A) male and (B) female.

(A)

|  |  |  | **Male** | | | | |
| --- | --- | --- | --- | --- | --- | --- | --- |
|  |  | **GGT points** | | | | | **p for interaction** |
|  |  | **0** | **1** | **2** | **3** | **4** |  |
| **Respiratory cancer** | | | | | | | |
| **Smoking** | None | 1(ref.) | 1.13 (0.97,1.32) | 1.41 (1.18,1.68) | 1.38 (1.15,1.67) | 1.46 (1.26,1.70) | 0.498 |
|  | Ex | 1(ref.) | 1.14(1.00,1.30) | 1.27 (1.09,1.48) | 1.15 (0.98,1.37) | 1.31 (1.16,1.48) |  |
|  | Current | 1(ref.) | 1.14 (1.04,1.26) | 1.21 (1.08,1.35) | 1.32 (1.19,1.47) | 1.40 (1.29,1.51) |  |
| **BMI** | <18.5 | 1(ref.) | 1.38 (0.92,2.08) | 1.68 (1.023,2.76) | 2.69(1.81,4.00) | 1.97(1.38,2.81) | 0.002 |
|  | 18.5≤BMI<25 | 1(ref.) | 1.16 (1.07,1.27) | 1.282(1.16,1.42) | 1.273(1.15,1.41) | 1.423(1.32,1.54) |  |
|  | 25≤BMI<30 | 1(ref.) | 1.09 (0.96,1.24) | 1.222(1.07,1.40) | 1.263(1.105,1.443) | 1.306(1.18,1.45) |  |
|  | ≥30 | 1(ref.) | 0.93 (0.58,1.47) | 0.835(0.50,1.41) | 0.899(0.55,1.46) | 1.101(0.76,1.59) |  |
| **GGT Quartile** | Q1 | 1(ref.) | 1.16 (0.89,1.52) | 1.79 (1.09,2.93) | 0.74 (0.28,1.98) | - | 0.055 |
|  | Q2 | 1(ref.) | 1.11 (0.95,1.29) | 1.07 (0.81,1.41) | 2.04 (1.50,2.78) | - |  |
|  | Q3 | 1(ref.) | 1.05 (0.94,1.16) | 1.16 (1.03,1.31) | 1.19 (1.03,1.37) | - |  |
|  | Q4 | 1(ref.) | - | 1.12 (0.94,1.32) | 1.11 (0.95,1.30) | 1.23 (1.07,1.41) |  |
| **Alcohol** | None | 1(ref.) | 1.05 (0.93-1.18) | 1.17(1.01-1.36) | 1.29 (1.10-1.51) | 1.35 (1.18-1.55) | 0.90 |
|  | Mild | 1(ref.) | 1.19(1.08-1.30) | 1.29 (1.08-1.43) | 1.31 (1.18-1.45) | 1.42 (1.31-1.53) |  |
|  | Heavy | 1(ref.) | 1.24(0.98-1.56) | 1.36 (0.98-1.72) | 1.29 (1.03-1.61) | 1.42 (1.19-1.68) |  |
| **Laryngeal cancer** | | | | | | | |
| **Smoking** | None | 1(ref.) | 1.58 (0.87,2.84) | 1.68 (0.83,3.43) | 2.18 (1.12,4.22) | 1.78 (1.00,3.19) | 0.9429 |
|  | Ex | 1(ref.) | 1.16 (0.73,1.84) | 1.53 (0.93,2.51) | 1.50 (0.90,2.47) | 1.57 (1.05,2.35) |  |
|  | Current | 1(ref.) | 1.33 (0.92,1.93) | 1.63 (1.102,2.397) | 1.54 (1.051,2.263) | 1.99 (1.50,2.65) |  |
| **BMI** | <18.5 | 1(ref.) | 5.58 (1.54,20.28) | 12.10 (3.54,41.35) | 4.41 (0.85,22.81) | 6.79 (2.02,22.91) | 0.021 |
|  | 18.5≤BMI<25 | 1(ref.) | 1.45 (1.05,1.99) | 1.75 (1.22,2.50) | 1.75 (1.23,2.50) | 1.80 (1.35,2.40) |  |
|  | 25≤BMI<30 | 1(ref.) | 0.98 (0.60,1.60) | 1.19 (0.71,2.00) | 1.39 (0.86,2.23) | 1.86(1.31,2.65) |  |
|  | ≥30 | 1(ref.) | 0.62 (0.13,2.91) | 0.37 (0.05,2.94) | 0.64 (0.13,3.06) | 0.52 (0.15,1.84) |  |
| **GGT Quartile** | Q1 | 1(ref.) | 0.74 (0.18,3.01) | 1.84 (0.256,13.33) | - | - | 0.70 |
|  | Q2 | 1(ref.) | 1.651(0.973,2.801) | 1.52 (0.56,4.13) | 2.68 (0.84,8.49) | - |  |
|  | Q3 | 1(ref.) | 0.894(0.6,1.33) | 1.18 (0.76,1.81) | 1.49 (0.93,2.37) | - |  |
|  | Q4 | 1(ref.) | - | 1.04 (0.59,1.80) | 0.88 (0.52,1.48) | 1.11 (0.71,1.75) |  |
| **Alcohol** | None | 1(ref.) | 1.05 (0.64,1.75) | 1.25 (0.67,2.33) | 0.94(0.43,2.02) | 1.26 (0.70,2.26) | 0.362 |
|  | Mild | 1(ref.) | 1.31 (0.94,1.84) | 1.54 (1.07,2.24) | 2.00 (1.44,2.78) | 2.03 (1.56,2.65) |  |
|  | Heavy | 1(ref.) | 2.05 (1.04,4.04) | 2.56(1.32,4.99) | 1.31 (0.61,2.79) | 2.15 (1.25,3.70) |  |
| **Lung cancer** | | | | | | | |
| **Smoking** | None | 1(ref.) | 1.108(0.942,1.302) | 1.39 (1.15,1.67) | 1.33 (1.09,1.62) | 1.45 (1.24,1.69) | 0.37 |
|  | Ex | 1(ref.) | 1.145(1,1.312) | 1.28 (1.07,1.47) | 1.12 (0.95,1.33) | 1.29 (1.13,1.46) |  |
|  | Current | 1(ref.) | 1.129(1.023,1.246) | 1.17 (1.05,1.31) | 1.31 (1.18,1.46) | 1.35 (1.25,1.47) |  |
| **BMI** | <18.5 | 1(ref.) | 1.31 (0.85,2.00) | 1.25 (0.70,2.21) | 2.68 (1.79,4.00) | 1.75 (1.20,2.55) | 0.007 |
|  | 18.5≤BMI<25 | 1(ref.) | 1.14 (1.05 ,1.25) | 1.25 (1.13,1.39) | 1.25 (1.12,1.39) | 1.40 (1.29,1.52) |  |
|  | 25≤BMI<30 | 1(ref.) | 1.10(0.96,1.25) | 1.22 (1.05,1.40) | 1.24 (1.08,1.43) | 1.26 (1.12,1.40) |  |
|  | ≥30 | 1(ref.) | 1.01 (0.63,1.64) | 0.90 (0.53,1.55) | 0.94 (0.56,1.57) | 1.19 (0.81,1.75) |  |
| **GGT Quartile** | Q1 | 1(ref.) | 1.18 (0.90,1.56) | 1.78 (1.07,2.96) | 0.79 (0.29,2.10) | - | 0.02 |
|  | Q2 | 1(ref.) | 1.07 (0.92,1.25) | 1.04 (0.78,1.39) | 1.99 (1.44,2.74) | - |  |
|  | Q3 | 1(ref.) | 1.06 (0.95,1.18) | 1.16 (1.02,1.31) | 1.17 (1.01,1.36) | - |  |
|  | Q4 | 1(ref.) | - | 1.12 (0.94,1.34) | 1.14 (0.97,1.34) | 1.24 (1.07,1.44) |  |
| **Alcohol** | None |  | 1.05 (0.93,1.19) | 1.17 (1.00 ,1.36) | 1.30 (1.11,1.53) | 1.36 (1.18,1.56) | 0.903 |
|  | Mild |  | 1.17 (1.07,1.29) | 1.27 (1.14,1.41) | 1.26 (1.13,1.39) | 1.37 (1.27,1.48) |  |
|  | Heavy |  | 1.19 (0.93,1.52) | 1.24 (0.96,1.60) | 1.31 (1.05,1.65) | 1.36 (1.13,1.62) |  |

(B)

|  |  | **Female** | | | | | |
| --- | --- | --- | --- | --- | --- | --- | --- |
|  | **GGT points** | | | | | | |
|  |  | **0** | **1** | **2** | **3** | **4** |  |
| **Respiratory tract** | | | | | | | |
| **Smoking** | None | 1(ref.) | 1.02 (0.88,1.18) | 0.88 (0.73,1.06) | 1.10 (0.92,1.32) | 0.96 (0.822,1.128) | 0.66 |
|  | Ex | 1(ref.) | 0.66 (0.07,5.98) | 1.58 (0.28,8.90) | 1.24 (0.21,7.42) | 1.77 (0.429,7.265) |  |
|  | Current | 1(ref.) | 0.66 (0.18,2.415) | 0.56 (0.121,2.61) | 1.34 (0.44,4.07) | 0.60 (0.2,1.801) |  |
| **BMI** | <18.5 | 1(ref.) | 1.20 (0.59,2.45) | 0.71 (0.22,2.34) | 2.45 (1.12,5.37) | 1.41 (0.58,3.45) | 0.51 |
|  | 18.5≤BMI<25 | 1(ref.) | 0.97 (0.81,1.15) | 0.92 (0.73,1.14) | 1.10 (0.88,1.37) | 1.00 (0.83,1.21) |  |
|  | 25≤BMI<30 | 1(ref.) | 1.02 (0.74,1.40) | 0.75 (0.51,1.12) | 0.98 (0.68,1.40) | 0.90 (0.67,1.21) |  |
|  | ≥30 | 1(ref.) | 2.07 (0.66,6.56) | 1.24 (0.33,4.65) | 1.45 (0.43,4.83) | 0.67 (0.21,2.12) |  |
| **GGT Quartile** | Q1 | 1(ref.) | 0.53 (0.30,0.96) | 1.73 (0.81,3.68) | - | - | 0.09 |
|  | Q2 | 1(ref.) | 1.22 (0.96,1.56) | 0.52 (0.28,0.99) | 1.29 (0.64,2.62) |  |  |
|  | Q3 | 1(ref.) | 1.00 (0.77,1.28) | 0.90 (0.64,1.25) | 1.21 (0.83,1.76) |  |  |
|  | Q4 | 1(ref.) | - | 0.96 (0.69,1.34) | 1.15 (0.84,1.56) | 1.03 (0.78,1.36) |  |
| **Alcohol** | None | 1(ref.) | 1.05 (0.89,1.24) | 0.923 (0.75,1.14) | 1.13 (0.92,1.38) | 1.01 (0.84,1.20) |  |
|  | Mild | 1(ref.) | 0.85 (0.61,1.19) | 0.74 (0.48,1.13) | 1.08 (0.74,1.58) | 0.85 (0.62,1.18) |  |
|  | Heavy | 1(ref.) | 0.43 (0.05,3.99) | - | - | - |  |
| **Laryngeal cancer** | | | | | | | |
| **Smoking** | None | 1(ref.) | 1.94 (0.32,11.73) | 1.63 (0.17,16.04) | 3.66 (0.59,22.93) | 6.21 (1.42,27.23) | 1 |
|  | Ex | 1(ref.) | - | - | - | - |  |
|  | Current | 1(ref.) | - | - | - | - |  |
| **BMI** | <18.5 | 1(ref.) | - | - | - | - | 0.99 |
|  | <25 | 1(ref.) | 8.69 (0.89,84.68) | 4.78 (0.29,78.21) | 5.68 (0.34,94.04) | 13.71 (1.44,130.51) |  |
|  | <30 | 1(ref.) | - | - | 2.79 (0.17,47.08) | 2.65 (0.21,32.92) |  |
|  | ≥30 | 1(ref.) | - | - | - | - |  |
| **GGT Quartile** | Q1 | 1(ref.) | 6.47 (0.58,72.55) | - | - | - | 1 |
|  | Q2 | 1(ref.) | - | - | - | - |  |
|  | Q3 | 1(ref.) | 3.36 (0.29,38.48) | 2.80 (0.16,48.57) | - | - |  |
|  | Q4 | 1(ref.) | - | - | - | - |  |
| **Alcohol** | None | 1(ref.) | 1.94 (0.32,11.73) | 1.63 (0.17,16.04) | 3.66 (0.59,22.93) | 6.21 (1.42,27.23) |  |
|  | Mild | 1(ref.) | - | - | - | - |  |
|  | heavy | 1(ref.) | - | - | - | - |  |
| **Lung cancer** | | | | | | | |
| **Smoking** | None | 1(ref.) | 1.01 (0.87,1.17) | 0.88 (0.72,1.06) | 1.09 (0.91,1.31) | 0.95 (0.81,1.11) | 0.75 |
|  | Ex | 1(ref.) | - | 1.64 (0.29,9.27) | 1.34 (0.23,8.02) | 1.88 (0.45,7.82) |  |
|  | Current | 1(ref.) | 0.66(0.18,2.42) | 0.56 (0.12,2.61) | 1.34 (0.44,4.07) | 0.60 (0.20,1.80) |  |
| **BMI** | <18.5 | 1(ref.) | 1.24 (0.61,2.53) | 0.74 (0.23,2.42) | 2.54 (1.16,5.58) | 1.46 (0.59,3.57) | 0.46 |
|  | <25 | 1(ref.) | 0.95 (0.80,1.13) | 0.91 (0.73,1.13) | 1.09 (0.87,1.36) | 0.99 (0.82,1.19) |  |
|  | <30 | 1(ref.) | 1.03 (0.75,1.41) | 0.76 (0.51,1.13) | 0.96 (0.67,1.38) | 0.88 (0.65,1.19) |  |
|  | ≥30 | 1(ref.) | 2.07 (0.66,6.56) | 1.24 (0.33,4.65) | 1.45 (0.43,4.83) | 0.67 (0.21,2.12) |  |
| **GGT Quartile** | Q1 | 1(ref.) | 0.49 (0.27,0.90) | 1.73 (0.81,3.69) | - | - | 0.06 |
|  | Q2 | 1(ref.) | 1.22 (0.96,1.56) | 0.52 (0.278,0.99) | 1.29 (0.64,2.62) | - |  |
|  | Q3 | 1(ref.) | 0.98 (0.758,1.27) | 0.88 (0.63,1.23) | 1.21 (0.83,1.77) | - |  |
|  | Q4 | 1(ref.) | - | 0.96 (0.69,1.35) | 1.13 (0.83,1.54) | 1.01 (0.77,1.34) |  |
| **Alcohol** | None | 1(ref.) | 1.05 (0.89,1.23) | 0.92 (0.75,1.14) | 1.11 (0.91,1.37) | 0.99 (0.83,1.18) | 0.968 |
|  | Mild | 1(ref.) | 0.83 (0.60,1.16) | 0.74 (0.48,1.13) | 1.09 (0.75,1.58) | 0.86 (0.62,1.19) |  |
|  | Heavy | 1(ref.) | 0.43 (0.05,3.99) | - | - | - |  |
